# Comparison of Dance and AAPM/EFOMP TG282 breast dosimetry methodologies for a screening population: Evaluation of model‐based median breast density metrics

**DOI:** 10.1002/acm2.70260

**Published:** 2025-09-24

**Authors:** Ioannis Delakis

**Affiliations:** ^1^ Biomedical Technology Services Queensland Health Brisbane Queensland Australia

**Keywords:** breast density, dosimetry, mammography dose, mean glandular dose, TG282

## Abstract

**Background:**

Accurate mean glandular dose (MGD) estimation is important in breast cancer screening programs to balance diagnostic benefit with radiation risk.

**Purpose:**

This study aimed to compare the performance of the Dance and AAPM/EFOMP Task Group 282 (TG282) breast dosimetry methodologies using model versus image‐derived breast density metrics.

**Methods:**

This study analyzed over 80,000 digital mammography images acquired in 2023 from BreastScreen Queensland (BSQ). Data were obtained from Siemens and Hologic systems and included 2D cranio‐caudal and mediolateral oblique views. Images with compressed breast thickness (CBT) between 20 and 100 mm were included. Volumetric breast density (VBD) and glandularity were extracted using Volpara software. MGD was estimated using both Dance and AAPM/EFOMP TG282 models, employing model‐based median and image‐measured breast density metrics. The ratios of MGD estimated using model medians to those using measured values ($R_{\mathrm{Dance}}$ and $R_{\mathrm{TG}282}$) were analyzed across CBT, and Pearson correlations (r) were computed.

**Results:**

The Dance model median glandularity overestimates population‐derived glandularity for most CBT, resulting in $R_{\mathrm{Dance}}$ > 1 at low CBT, with the trend reversed for CBT > 80 mm. $R_{\mathrm{Dance}}$ showed moderate positive correlation with CBT (*r* = 0.57 Hologic; *r* = 0.63 Siemens, *p *< 0.001). $R_{\mathrm{TG}282}$ remained close to unity across CBT, with weak negative correlations (*r* = −0.17 Hologic; *r* = −0.04 Siemens, *p* < 0.001), indicating consistency between model and measured VBD.

**Conclusions:**

The AAPM/EFOMP TG282 dosimetry model exhibited stronger agreement between median model‐predicted and population‐specific measured breast density metrics than the Dance model. This resulted in improved consistency in ratios of estimated MGD values based on median model‐to‐measured breast density metrics across the full range of CBT, when using the AAPM/EFOMP TG282 methodology.

## 1 INTRODUCTION

Mean glandular dose (MGD) refers to the average radiation dose absorbed by the glandular tissue of the breast and serves as the primary metric for assessing radiation risk in mammography. MGD is a key parameter used extensively in mammography for protocol optimization, regulatory compliance testing, and quality control. Accurate quantification of MGD is particularly important in breast screening programmes, where large numbers of asymptomatic individuals are exposed to ionizing radiation, in order to appropriately balance the associated risks and benefits.

Over the past few decades, three main methodologies have been developed to quantify MGD: Wu,^1^, ^2^ Boone,^3^, ^4^ and Dance.^5^, ^6^ Although the Dance method has been widely adopted by several groups—including the International Atomic Energy Agency (IAEA),^7^ American College of Radiology (ACR),^8^ United Kingdom,^9^ and Australia^10^—it is not without limitations, which have been extensively discussed in prior work.^11^ In summary, these limitations include its reliance on cranio‐caudal (CC) projections over medio‐lateral oblique (MLO) views, the need for continual updates to accommodate new anode/filter combinations, and its simplified approach for estimating MGD for more advanced and complex imaging studies such as digital breast tomosynthesis and contrast‐enhanced mammography.

As our understanding of breast anatomy and population‐based breast density distributions has evolved, the limitations of the Dance methodology have become increasingly apparent. In response, the American Association of Physicists in Medicine (AAPM) and the European Federation for Organisations of Medical Physics (EFOMP) Task Group 282 (TG282) initiated efforts to develop an updated and more robust dosimetry model.^12^ This incorporated anatomically realistic breast models based on breast computed tomography (CT) population‐based data, enabling more accurate representation of fibroglandular tissue distribution and breast shapes, including CC and MLO views. It also included detailed modeling of skin thickness, pectoral muscle, and the x‐ray heel effect, leading to more precise dose estimates.

Recent studies comparing the Dance with the AAPM/EFOMP TG282 methodology using data from a breast screening programme have reported an average reduction in the calculated MGD of approximately 20% for digital mammography (DM) when using the AAPM/EFOMP TG282 method.^13^ Building on this work, the present study extends the comparison by assessing the degree of concordance between model‐derived median breast density values and population‐specific breast density statistics and by attempting to quantify the uncertainty in calculated MGD values resulting from the use of median model‐based densities for each methodology. Given that personalized, quantitative breast density metrics are typically not readily available in clinical settings, this study provides valuable insights into differences between the Dance and AAPM/EFOMP TG282 dosimetry models.

## 2 METHODS

Data were collected from BreastScreen Queensland (BSQ), the state‐wide breast screening program operating in Queensland, Australia. Each BSQ site included in this study was equipped with DM units of two different vendors: Siemens Healthineers GmbH (Forchheim, Germany) and Hologic inc. (Bedford, MA, USA). The Siemens MAMMOMAT Revelation DM units use an anode/filter combination of Tungsten/Rhodium (W/Rh) for all compressed breast thickness (CBT), while the Hologic 3Dimensions and Selenia Dimensions systems use W/Rh for CBT up to 70 mm, above which they switch to an anode/filter combination of Tungsten/Silver (W/Ag), in accordance with the unit's automatic exposure control functionality.

The study included 2D views, both CC and MLO, performed over the period of January to December 2023. Studies of CBT outside the range of 20 to 100 mm were excluded as they were deemed atypical for our screening population. Additionally, magnification and tomosynthesis 2D reconstructed views, and images with breast implants were excluded.

Mammography images were analyzed using Volpara software, version 3.4 (Volpara Health Technologies, Wellington, New Zealand). Volpara uses image data information and modeling to calculate the fibroglandular and adipose tissues that would result in the measured signal level at each detector element location.^14^ Two key breast density metrics were extracted for each image using Volpara: glandularity (g) by mass (%)^15^ and volumetric breast density (VBD), defined as the volumetric ratio of fibroglandular tissue to total breast volume (%).^12^ Prior work has demonstrated cross‐vendor consistency in glandularity and VBD measurements when using Volpara.^16^ The g and VBD were used in the estimation of MGD using the Dance and TG282 methodologies, respectively. All other parameters required for the estimation of MGD were extracted from image DICOM headers, including half‐value layer (HVL), tube kilovoltage peak (kVp), anode/filter material combination, Air Kerma (AK), and the age of the screening client. It should be noted that this study evaluates MGD ratios rather than absolute MGD values. As a result, AK values are normalized in the ratio calculations presented in the following paragraphs, and no correction to the reference point of the TG282 methodology was required.^12^ The distributions of measured g and VBD were also characterized by calculating the median and median absolute deviation (MAD) values of data for each CBT.

For each image, MGD was estimated in the following ways:

1. Using the median population value of the breast density metric as provided in each methodology. This yielded $\mathrm{MG}D_{\mathrm{Dance}-\mathrm{median}}$ and $\mathrm{MG}D_{\mathrm{TG}282-\mathrm{median}}$ values, for Dance and AAPM/EFOMP TG282 methodologies, respectively.
2. Using the values of breast density metrics as measured for each image using Volpara. This yielded $\mathrm{MG}D_{\mathrm{Dance}-\mathrm{measured}}$ and $\mathrm{MG}D_{\mathrm{TG}282-\mathrm{measured}}$, for Dance and AAPM/EFOMP TG282 methodologies, respectively.

Following this, and for each methodology, the ratio of the MGD obtained using the model's median density metric to the MGD obtained using the measured breast density metric was estimated as:

(1) $$R_{\mathrm{Dance}}=\frac{\mathrm{MG}D_{\mathrm{Dance}-\mathrm{median}}}{\mathrm{MG}D_{\mathrm{Dance}-\mathrm{measured}}}$$

and

(2) $$R_{\mathrm{TG}282}=\frac{\mathrm{MG}D_{\mathrm{TG}282-\mathrm{median}}}{\mathrm{MG}D_{\mathrm{TG}282-\mathrm{measured}}}$$

The distribution of the ratios $R_{\mathrm{Dance}}$ and $R_{\mathrm{TG}282}$ across different CBT values were then analyzed using Pearson correlation to assess the agreement between using median model values versus measured values for MGD calculation in our screening population. This included calculation of the Pearson correlation coefficient (*r*) and the associated *p*‐value using a *t*‐test for statistical significance.

## 3 RESULTS

**FIGURE 1:**
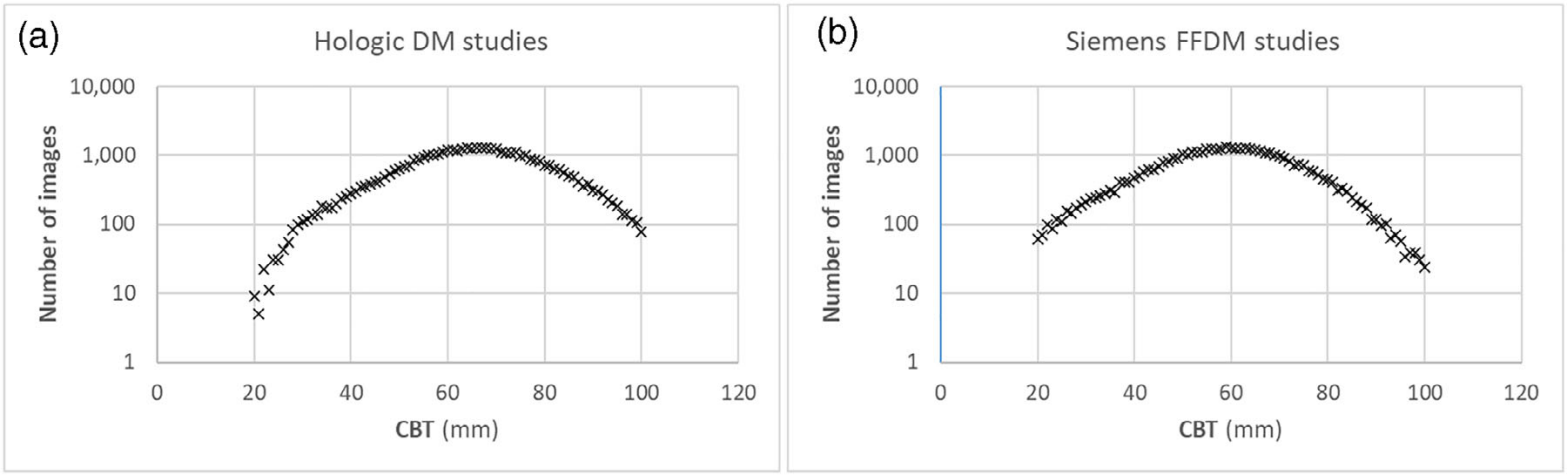
Distribution of image data over different CBT for Hologic (a) and Siemens (b) DM studies.

The dataset included over 80,000 images, with a well‐balanced representation of DM systems from the two vendors, as detailed in Table 1. The median age of BSQ clients was 60 years. As illustrated in Figure 1, the distribution of data across different CBT followed a similar pattern for both vendor datasets, although for Hologic DM units there were more images for large CBT and much fewer images for smaller CBT, compared to Siemens DM units.

**TABLE 1:** Number of images per DM system at each BSQ site used in this study. “None” indicates that the vendor/model of the DM system is not available at the BSQ site location.

BSQ site location	Siemens MAMMOMAT Revelation	Hologic 3Dimensions	Hologic Selenia Dimensions
Chermside, North Brisbane	6,578	10,140	None
Queen Elisabeth II, South Brisbane	14,455	None	8,492
Ipswich	10,175	None	4,308
Townsville	7,243	9,181	None
Mackay	6,578	None	13,063

**FIGURE 2:**
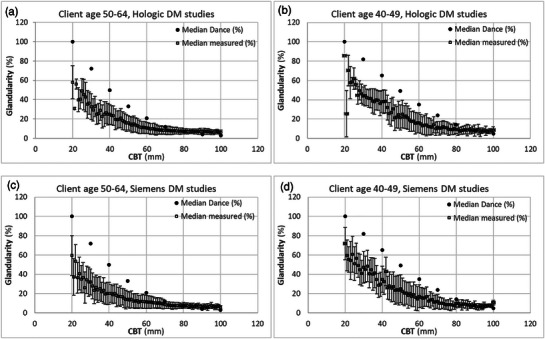
Median glandularity derived from our image dataset against median glandularity given by the Dance model across different CBT. Data shown for Hologic DM and breast screening client ages of 50–64 (a) and 40–49 (b); and for Siemens DM and breast screening client ages of 50–64 (c) and 40–49 (d). Error bars represent the median absolute deviation of measured data for each CBT.

**FIGURE 3:**
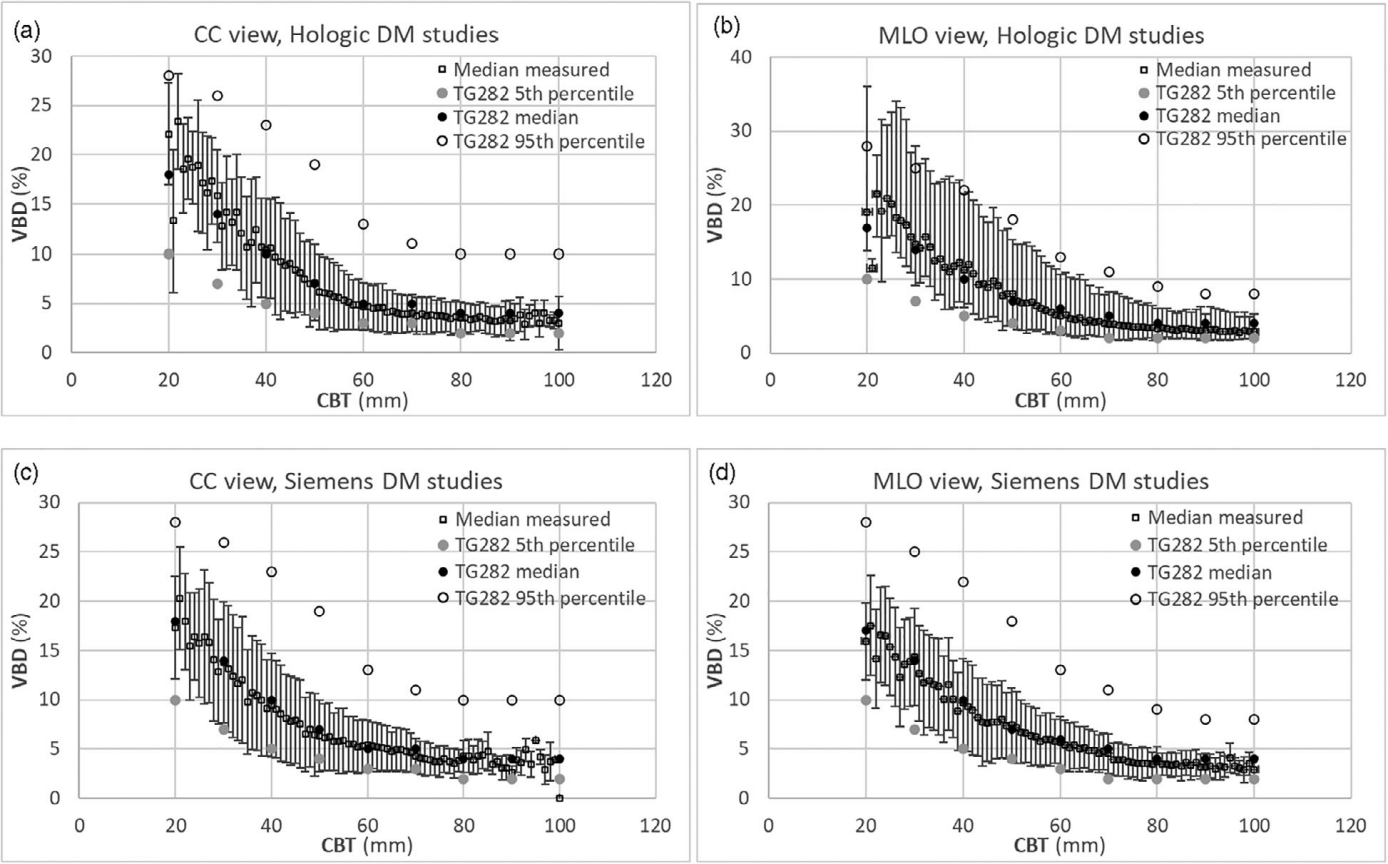
Median VBD derived from our image dataset against 5th, 50th (Median) and 95th percentile VBD values adopted by the AAPM/EFOMP TG282 breast model. Data shown for Hologic DM and CC (a) and MLO (b) views, and for Siemens DM and CC (c) and MLO (d) views. Error bars represent the median absolute deviation of measured data for each CBT.

Figure 2 compares the median glandularity used in the Dance breast model with the median glandularity by mass derived from our dataset, differentiated by age groups as per Dance methodology. Figure 3 presents the 5th, 50th (median), and 95th percentile VBD values from the AAPM/EFOMP TG282 breast model alongside the median VBD from our dataset, differentiated between CC and MLO views. The agreement between the median VBD from our dataset and the median VBD from the TG282 model is overall high with a deviation observed for the Hologic dataset in the 20–30 mm CBT range, where the measured median VBD slightly exceeded the TG282 reference values.

**FIGURE 4:**
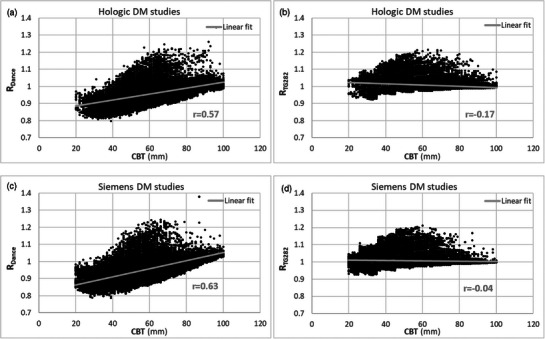
Distribution of R_Dance_ (a) and R_TG282_ (b) across different CBT for Hologic DM dataset; and distribution of R_Dance_ (c) and R_TG282_ (d) across different CBT for Siemens DM dataset. Pearson correlation coefficient (*r*) is also shown for each distribution (*p* < 0.001 for all correlations).

Figure 4 describes the distributions of $R_{\mathrm{Dance}}$ and $R_{\mathrm{TG}282}$ across CBT for the different DM vendor units. The Pearson correlation coefficients (*r*) between $R_{\mathrm{Dance}}$ and CBT were 0.57 (*p* < 0.001) for Hologic (Figure 4a) and 0.63 (*p* < 0.001) for Siemens (Figure 4c), indicating a moderate positive correlation. The Pearson correlation coefficients between $R_{\mathrm{TG}282}$ and CBT were −0.17 (*p* < 0.001) for Hologic (Figure 4b) and −0.04 (*p* < 0.001) for Siemens (Figure 4d), indicating very weak negative correlations and suggesting no strong relationship or tendency in the $R_{\mathrm{TG}282}$ across CBT.

## 4 DISCUSSION

As shown in Figure 2, comparison of the median glandularity of the Dance breast model with the median glandularity measured in our population reveals an overestimation of the model's median glandularity across most CBT for both Hologic and Siemens DM systems. This trend only reverses marginally for CBT over 80 mm, where the model slightly underestimates median glandularity relative to the population‐derived values, congruent with previously published results.^17^ These findings demonstrate how the Dance methodology overestimates breast glandularity, primarily on account of basing estimates biased toward the densest breast regions and assuming a uniform glandular tissue distribution.^11^, ^12^, ^13^

On the other hand, Figure 3 illustrates the closer agreement between the median VBD from the AAPM/EFOMP TG282 model and VBD values computed from our study population. The deviation for the Hologic dataset in the 20–30 mm CBT range is likely attributable to the small sample size within this CBT subset of the Hologic DM systems, as also shown in Figure 1, leading to reduced statistical robustness of computed values.

The positive correlation in Figures 4a,c indicates that MGD is lower when estimated using the Dance breast model's median glandularity compared to when using measured g values. The underestimation is most pronounced at the lower end of the CBT spectrum, with the pattern reversed for breast thicknesses over 80 mm. This can be explained with reference to Figure 2, which shows that the Dance model assumes overall a higher median glandularity than that measured in our population data; this overestimation leads to lower MGD estimates for a given entrance AK, and conversely, underestimation would result in higher MGD values. The findings from this study corroborate with earlier work conducted on different beam qualities and with a smaller dataset.^17^ For large CBT we can also observe a “flattening‐out” of $R_{\mathrm{Dance}}$ for the dataset from Hologic systems (Figure 4a) compared to the dataset for the Siemens systems (Figure 4c). This can be explained by the fact that Hologic systems use a harder beam than Siemens for large CBT: the small differences between Dance and measured glandularities will make more of a difference to estimated MGD for the softer beam quality used by Siemens.

The low Pearson correlation coefficients between $R_{\mathrm{TG}282}$ and CBT shown in Figures 4b,d indicate a close agreement between MGD estimated using the AAPM/EFOMP TG282 model's median VBD and MGD estimated using measured VBD values. $R_{\mathrm{TG}282}$ — defined in formula (2) as the ratio of MGD estimated using the AAPM/EFOMP TG282 model's VBD to that estimated using image‐specific measured VBD — was generally much closer to unity and more consistent across CBT for both Hologic and Siemens systems compared to $R_{\mathrm{Dance}}$. The slightly weak negative correlation for the Hologic dataset is likely due to the small number of studies with small CBT that had relatively dense breasts, leading to a higher measured VBD and thus a lower $\mathrm{MG}D_{\mathrm{TG}282-\mathrm{measured}}$, resulting in a ratio greater than unity.

Our findings demonstrate that the AAPM/EFOMP TG282 methodology, having adopted a more sophisticated breast model, is less susceptible to potential inaccuracies arising from the use of generic breast density assumptions. Although our work used individualized breast density metrics, it is worth noting that the MGD estimated using either the Dance or the AAPM/EFOMP TG282 model are not intended to be applicable to individuals but rather represent the average dose to a typical breast.

A key limitation of the analysis presented in this article is that it is limited to DM systems from two vendors and the population of Queensland is of predominantly European ancestry. Future investigations involving populations of different ancestry and using datasets from different DM systems may provide additional insights.

## 5 CONCLUSION

This study evaluates the appropriateness of model‐based median breast densities for a specific population and compares the resulting uncertainty in MGD estimates when using the Dance and AAPM/EFOMP TG282 dosimetry models. Our findings demonstrate that the Dance model tends to overestimate glandularity across most CBT particularly in thinner breasts. On the other hand, the AAPM/EFOMP TG282 model exhibited stronger concordance between model‐predicted and measured VBD. This led to improved consistency in the ratio of MGD estimates derived from model‐predicted to measured VBD across the full range of CBT. The AAPM/EFOMP TG282 methodology, with its refined breast model and VBD‐based approach, was less sensitive to deviations introduced by population‐specific density characteristics and beam quality variations. These results support the robustness and adaptability of AAPM/EFOMP TG282 over the Dance breast dosimetry model.

## AUTHOR CONTRIBUTIONS

The author conceived and designed the project and conducted the data collection and analysis. The manuscript was also written and submitted by the author.

## CONFLICT OF INTEREST STATEMENT

The author declares no conflict of interest.
